# Comparison of egg-shape equations using relative curvature measures of nonlinearity

**DOI:** 10.1016/j.psj.2024.104069

**Published:** 2024-07-10

**Authors:** Meng Lian, Ke He, David A. Ratkowsky, Long Chen, Jinfeng Wang, Lin Wang, Weihao Yao, Peijian Shi

**Affiliations:** ⁎ Department of Applied Mathematics, College of Science, Nanjing Forestry University, Nanjing 210037, China; † School of Architecture, Huaqiao University, Xiamen 361021, China; ‡ Tasmanian Institute of Agriculture, University of Tasmania, Hobart, Tasmania 7001, Australia; § Bamboo Research Institute, College of Ecology and Environment, Nanjing Forestry University, Nanjing 210037, China

**Keywords:** egg volume, goodness-of-fit, parameter-effects curvature, Preston equation, Troscianko equation

## Abstract

A 2-dimensional (**2D**) egg-shape equation can be used to construct a 3D egg geometry based on the hypothesis that an egg is a solid of revolution, which helps to calculate egg volume and surface area. The parameters in the 2D egg-shape equation are potentially valuable for providing a clue to the ecology and evolution of avian eggs. In this study, the 5-parameter Preston equation (**PE**), the 4-parameter Troscianko equation (**TE**), and another 2 egg-shape equations, were compared in describing real 2D egg-shape data of 300 *Gallus gallus domesticus* eggs and additional 50 eggs that represented the variation in avian egg geometries. Adjusted root-mean-square error was used to quantify each equation's prediction error. Given that the 4 equations are nonlinear, relative curvature measures of nonlinearity were used to assess the extent of nonlinearity in each equation. PE was found to be the best among the 4 equations in terms of adjusted root-mean-square error and minimizing nonlinearity. The empirically determined egg volumes using a graduated cylinder were compared with the predicted egg volumes using the formula for a solid of revolution based on 2D predictions from the 4 egg-shape equations. There were negligible differences in the predicted egg volumes and surface areas among the 4 equations, indicating that these equations are all valid in calculating egg volume and surface area. In addition, we proposed a 5-parameter TE and found that it outperformed the above 4 equations in describing the 2D egg shape of *G. gallus*, but was less general than PE for other egg shapes. This work provides statistical evidence to show which equation is the best for describing the geometry of avian eggs and nondestructively calculating their volume and surface area, helping to classify poultry eggs into different grades according to the morphological characteristics of the eggs.

## INTRODUCTION

The shapes of avian eggs vary greatly from the spherical eggs of hawk-owl to the pyriform eggs of murre. There are many hypotheses to account for the various shapes. For example, the shape of avian eggs can be influenced by the pressure exerted by the fallopian tubes (Todd and Smart, 1984) and is also affected by the clutch size and the flying ability of female birds (Stoddard et al., 2017). Egg shape also relates to the efficiency of temperature utilization. An example is the orientation of the egg with respect to the incubation surface of the parents, i.e., the brood patch area lacking down feathers, which influences the efficiency of female incubation (Barta and Székely, 1997). In addition, during flight, gravid female birds need to package their eggs as tightly as possible in their bodies to minimize the impact on their flight. Consequently, birds with stronger flight ability tend to have longer or sharper eggs (Stoddard et al., 2019). Most pointed eggs are laid by birds usually nesting on a cliff, because that egg shape helps prevent the egg from rolling down the cliff. Nishiyama (2012) explains why this happens from a physical and mathematical perspective. The stable inclination of the midline of the egg due to the deviation of the center of mass will eventually cause the egg to rest on the sloping surface and stop rolling away. An appropriate egg shape is necessary for birds to survive and reproduce under the various constraints of their living environment and physiological conditions. A mathematical equation that accurately describes the profile of an avian egg is helpful to account for some physiological and mechanical reasons for egg shape formation since egg shape can be deemed as a response to the pressure exerted by the fallopian tubes during the formation of the eggshell (Thompson, 1917; Mallock, 1925; Todd and Smart, 1984).

If there are mathematical formulae that can accurately describe an egg's profile, the volume and surface area of the egg can be nondestructively calculated based on the formula for a solid of revolution, and the egg's 3-dimensional (**3D**) geometry can be obtained from its 2-dimensional (**2D**) image. This is helpful for researchers to better understand the ecology and evolution of eggs, and the artificial incubation for protected birds (Zlatev et al., 2022). Besides, it can also be used in industry, art, and architecture, e.g., egg-shaped sludge grinders, egg-shaped waterways, and ovoid carriages (Petrović et al., 2011; Regueiro-Picallo et al., 2016). Therefore, finding an accurate egg-shape equation is important and meaningful in many respects.

Previous studies devoted to model comparison that have proposed and compared equations for egg shape (e.g., Biggins et al., 2018, 2022; Shi et al., 2022a; and references therein) focused on the comparison of the goodness of fit (e.g., the root-mean-square error) or the trade-off between goodness of fit and model structural complexity (e.g., the Akaike information criterion). However, measures of nonlinearity which quantify the extent of nonlinear behavior in regression models and examine whether such models exhibit “close-to-linear” behavior (Ratkowsky, 1983) were rarely considered. In the present study, 4 representative egg-shape equations, namely the 5-parameter Preston equation, 4-parameter Preston equation, 4-parameter Troscianko equation, and 4-parameter Narushin-Romanov-Griffin equation (Preston, 1953; Troscianko, 2014; Narushin et al., 2021; Shi et al., 2023), were compared based on the adjusted root-mean-square error and the relative curvature measures of nonlinearity (Bates and Watts, 1980). In addition, a 5-parameter Troscianko equation was proposed and compared with the 5-parameter Preston equation.

## MATERIALS AND METHODS

### Models

Preston (1953) proposed a unified mathematical equation to describe any 2D profile of avian eggs, and found that 3 to 5 parameters were sufficient to describe the shapes of existing eggs:

(1) $$\left\{ \begin{array}{c} \;y_{P}=a\sin\theta \\ x_{P}=b\cos\theta \left(1+c_{1}\sin\theta +c_{2}\sin^{2}\theta +c_{3}\sin^{3}\theta \right) \end{array}\right.$$

where *x_P_* and *y_P_* are the abscissa and ordinate coordinates, respectively, of an egg's 2D profile when the egg's midline is aligned with the *y*-axis; θ is the eccentric angle; *a* is half the length of the egg, and *b* is approximately half the maximum width of the egg; *c*_1_, *c*_2_, and *c*_3_ are additional parameters to be estimated.

However, Eq. (1) does not provide a direct functional relationship between the abscissa and ordinate coordinates of an egg's profile*.* Shi et al. (2023) re-expressed the Preston equation (denoted as **PE**) in a more explicit form in the Euclidean coordinate system:

(2) $$y_{1}=\pm b\sqrt{1-{\left(\frac{x}{a}\right)}^{2}}\cdot \left(1+c_{1}\frac{x}{a}+c_{2}{\left(\frac{x}{a}\right)}^{2}+c_{3}{\left(\frac{x}{a}\right)}^{3}\right)$$

where *x* = *y_P_, y*_1_ = *x_P_*. Due to the exchange of the abscissa and ordinate coordinates compared with Eq. (1), the midline of the egg's 2D profile is aligned with the *x*-axis in Eq. (2), and the egg base is on the right side of the *x*-axis. The model parameters are the same as those in Eq. (1). The positive and negative symbols on the right-hand side of Eq. (2) represent the upper and lower parts of the 2D profile about the *x*-axis, respectively.

PE can be further simplified for some egg shapes. Preston (1953) defined the shape generated by Eq. (2) without the parameter *c*_3_ as the “standard avian egg shape”, which can depict the geometries of many common egg shapes apart from pyriform eggs:

(3) $$y_{2}=\pm b\sqrt{1-{\left(\frac{x}{a}\right)}^{2}}\cdot \left(1+c_{1}\frac{x}{a}+c_{2}{\left(\frac{x}{a}\right)}^{2}\right).$$

We refer to Eq. (3) as the simplified Preston equation (**SPE**) hereinafter.

Troscianko (2014) asserted that the shape of an avian egg results from the intrinsic pressure variations in the oviduct, and assumed that the pressure is normally distributed along the egg's long axis (i.e., the midline of the egg). Thus, he multiplied the normal distribution function by the equation of the circle to obtain an egg's 2D equation. Biggins et al. (2022) rewrote the original egg-shape equation proposed by Troscianko (2014) as follows:

(4) $$y_{T}=\pm \text{exp}\left(\alpha_{0}+\alpha_{1}x_{T}+\alpha_{2}{x_{T}}^{2}\right)\sqrt{1-{x_{T}}^{2}}$$

where *x_T_* and *y_T_* represent the abscissa and ordinate, respectively, of an arbitrary point on the Troscianko curve, a scaled egg-shape curve where the egg length is equal to 2; α_0_, α_1_, and α_2_ are parameters to be estimated. In Eq. (4), the given *x_T_* values range between −1 and 1. The midline of the egg's 2D profile is also aligned with the *x-*axis. To express the functional relationship between the abscissa and ordinate of the actual egg shape more explicitly, we re-expressed Eq. (4) as follows:

(5) $$y_{3}=\pm a\text{exp}\left(\alpha_{0}+\alpha_{1}\left(\frac{x}{a}\right)+\alpha_{2}{\left(\frac{x}{a}\right)}^{2}\right)\sqrt{1-{\left(\frac{x}{a}\right)}^{2}}$$

where *x* and *y*_3_ represent the abscissa and ordinate, respectively, of the actual egg shape; *a* is half the length of the egg; α_0_, α_1_, and α_2_ are parameters to be estimated, which are the same as those in Eq. (4). We refer to Eq. (5) as **TE** for simplicity hereinafter.

Narushin et al. (2021) found that Hügelschäffer's equation (Petrović et al., 2011) is valid for describing 3 common egg shapes (i.e., circular, elliptical, and oval); however, it does not describe pyriform eggs well due to the limitations of its mathematical expression. Narushin et al. (2021) provided a general expression of Hügelschäffer's equation as follows:

(6) $$y_{4}=\pm f_{1}\left(x\right)\cdot \left\{1-E\left[1-f_{2}\left(x\right)\right]\right\},$$

where

(7) $$f_{1}\left(x\right)=\frac{B}{2}\sqrt{\frac{A^{2}-4x^{2}}{A^{2}+8Cx+4C^{2}}}$$

(8) $$f_{2}\left(x\right)=\sqrt{\frac{A\left(A^{2}+8Cx+4C^{2}\right)}{2\left(A-2C\right)x^{2}+\left(A^{2}+8AC-4C^{2}\right)x+2AC^{2}+A^{2}C+A^{3}}}$$

(9) $$E=\frac{\sqrt{5.5A^{2}+11AC+4C^{2}}\cdot \left(\sqrt{3}AB-2D\sqrt{A^{2}+2AC+4C^{2}}\right)}{\sqrt{3}AB\left(\sqrt{5.5A^{2}+11AC+4C^{2}}-2\sqrt{A^{2}+2AC+4C^{2}}\right)}$$

and *x* and *y*_4_ represent the abscissa and ordinate, respectively, of an egg's 2D profile in Euclidean coordinates; *A* is the length of the egg (i.e., *A* = 2*a*); *B* is the maximum width of the egg; *C* is equal to (*A−B*)/2*q*, in which *q* is a positive number to be estimated; and *D* is the egg's width associated with (3/4)*A* from the egg base to the egg tip on the midline of the egg's 2D profile. We refer to Eq. (9) as the **NRGE** for simplicity hereinafter.

In the present study, we used PE, SPE, TE, and NRGE to fit the real egg-shape data, because these equations were found to be more valid than the existing equations (Biggins et al., 2022; Shi et al., 2023).

### Calculation of Egg Surface Area and Volume

Since an egg is a solid of revolution, as proven by previous studies, and its 2D profile is axisymmetric (Paganelli et al., 1974; Shi et al., 2023), the surface area (***S***) and volume (***V***) of an egg can be approximated by revolving the egg's 2D profile. The calculation formulae of *S* and *V* are as follows:

(10) $$S=2\pi \int_{-a}^{a}y\sqrt{1+{\left(\frac{dy}{dx}\right)}^{2}}dx$$

and

(11) $$V=\pi \int_{-a}^{a}y^{2}dx$$

where *dy*/*dx* is the derivative of *y* with respect to *x; y* can be *y*_1_, *y*_2_, *y*_3,_ or *y*_4_ in Eqs. (2), (3), (5), and (6), respectively. When calculating *S*, the first-order partial derivatives for each of the 4 equations are required. In the present study, the first-order partial derivative formulae for the 4 equations are as follows, respectively:

(12) $$\frac{dy_{1}}{dx}=\frac{b\left[a^{4}c_{1}+a^{3}\left(2c_{2}-1\right)x+a^{2}\left(3c_{3}-2c_{1}\right)x^{2}-3ac_{2}x^{3}-4c_{3}x^{4}\right]}{a^{4}\sqrt{a^{2}-x^{2}}}$$

(13) $$\frac{dy_{2}}{dx}=\frac{b\left[a^{4}c_{1}+a^{3}\left(2c_{2}-1\right)x-2a^{2}c_{1}x^{2}-3ac_{2}x^{3}\right]}{a^{4}\sqrt{a^{2}-x^{2}}}$$

(14) $$\frac{dy_{3}}{dx}=\left\{\alpha_{1}+\frac{2\alpha_{2}}{a}x-\frac{x}{a}{\left[1-{\left(\frac{x}{a}\right)}^{2}\right]}^{-1}\right\}e^{\left\{\alpha_{0}+\alpha_{1}\left(\frac{x}{a}\right)+\alpha_{2}{\left(\frac{x}{a}\right)}^{2}\right\}}\sqrt{1-{\left(\frac{x}{a}\right)}^{2}}$$

(15) $$\frac{dy_{4}}{dx}=-\frac{4f_{1}\left(x\right)\left[Cf_{3}\left(x\right)+xf_{4}\left(x\right)\right]}{f_{3}\left(x\right)f_{4}\left(x\right)}\cdot \left\{1-E\left[1-f_{2}\left(x\right)\right]\right\}-\frac{AE}{2}\frac{f_{1}\left(x\right)}{f_{2}\left(x\right)}\frac{f_{4}\left(x\right)\left(2Fx+G\right)}{{\left(Fx^{2}+Gx+H\right)}^{2}}$$

where *f*_1_(x), *f*_2_(x) and *E* are defined by Eqs. (7) – (9), respectively, and

(16) $$f_{3}\left(x\right)=A^{2}-4x$$

(17) $$f_{4}\left(x\right)=A^{2}+8Cx+4C^{2}$$

(18) F=2(A−2C)

(19) $$G=A^{2}+8AC-4C^{2}$$

(20) $$H=2AC^{2}+A^{2}C+A^{3}$$

### Statistics and Tests Used for Model Comparison

*Root-mean-square error.* To assess the goodness of fit for the 4 equations, the root-mean-square error (RMSE) was calculated by comparing the predicted *V* (denoted as $\hat{V}$) with the observed *V* (denoted as *V*) empirically determined using a 500 mL glass graduated cylinder (diameter: 6 cm).

(21) $$\text{RMSE}\;=\sqrt{\frac{\sum_{i=1}^{n}{\left(V_{i}-{\hat{V}}_{i}\right)}^{2}}{n}}$$

where the subscript *i* represents the *i*-th egg, and *n* represents the number of eggs.

Additionally, the adjusted RMSE (**RMSE_adj_**) between the predicted and observed *y* values (i.e., the ordinate of the egg's 2D profile in the plane) was employed to compare the goodness of fit, and the purpose of the adjustment is to eliminate the influence of different egg sizes (Shi et al., 2023).

(22) $$\text{RMS}E_{\text{adj}}=\frac{\sqrt{\text{RSS}/N}}{W/2}$$

where RSS represents the residual sum of squares of the observed and predicted *y* values, *N* represents the number of data points on the egg's 2D profile, and *W* is the maximum width of the egg.

*Relative curvature measures of nonlinearity.* As mentioned earlier, nonlinear least-squares was used to fit the 4 egg-shape equations. Linear regression models, i.e., models where the parameters to be estimated appear linearly, have least squares estimators with optimal properties, producing unbiased, normally distributed, minimum variance estimates when the errors, embodied in the stochastic assumption, are independent and identically normally distributed (Ratkowsky, 1983). However, the parameter estimators of nonlinear regression models do not possess these so-called “asymptotic” properties in finite samples (Jennrich, 1969), and sometimes very large sample sizes are required to closely approximate the aforementioned properties in practice. Indeed, it is usually difficult to define what is meant by a “large” sample size. However, there are some model/data set combinations that may require only a small sample size for the asymptotic properties to be closely approximated, and these can be labeled “close to linear” (Ratkowsky, 1983). Some characteristics, such as parameter confidence intervals, prediction intervals of the dependent variable, the true correlation coefficients between parameters, and meaningful hypothesis tests can only be accurately achieved with parameter estimates that possess or at least closely approximate these close-to-linear properties. One approach to quantify the degree of nonlinearity is to use the relative curvature measures of nonlinearity proposed by Bates and Watts (1980). A concise discussion of why, when, and how to use these curvature measures has been presented by Karolczak (1995). Essentially, there are 2 components to the relative curvature measures: a root-mean-square intrinsic curvature $\gamma_{\mathrm{RMS}}^{N}$ and a root-mean-square parameter-effects curvature $\gamma_{\mathrm{RMS}}^{T}$ (Bates and Watts, 1980, 1988). The smaller the values of $\gamma_{\text{RMS}}^{N}$ and $\gamma_{\text{RMS}}^{T}$, the closer-to-linear is the model.

A nonlinear model with small relative curvatures has many advantages, such as reducing the number of fitting iterations, realizing fast parameter convergence, obtaining asymptotically unbiased parameter estimates, getting estimate parameters with asymptotic minimum variance, and efficiently applying linear model tests (e.g., *t*-tests) (Bates and Watts, 1988; Karolczak, 1995). Relative curvature measures of nonlinearity can help to decide between competing nonlinear models. For a fit of a specific nonlinear model, the relative curvature measures of nonlinearity have a critical value, $K_{c}\;=\;1/\sqrt{F\;(p,\;n\;-\;p,\;\alpha )}$, where *p* represents the number of model parameter(s), *n* represents the number of observations, and α represents the significance level, below which the linear approximation of the model is reasonable. However, because the 4 studied egg-shape equations do not all have the same number of parameters, their critical curvatures are not all the same. To diminish the influence of unequal parameter numbers, the ratio (denoted by **δ**) of the relative curvature to the corresponding critical curvature was calculated for each of the 4 equations. In contrast to the intrinsic curvature, which is not altered by reparameterization (i.e., parameter transformation), the parameter-effects curvature can be effectively reduced by appropriate reparameterization (Bates and Watts, 1980; Karolczak, 1995; Ratkowsky and Reddy, 2017). Thus, the root-mean-square parameter-effects curvature is a more suitable choice than the root-mean-square intrinsic curvature for the numerator of δ.

(23) $$\delta \;=\;\frac{\text{Root}-\text{mean-square}\;\text{parameter-effects}\;\text{curvature}}{\text{Critical}\;\text{curvature}}\;=\;\frac{\gamma_{\text{RMS}}^{T}}{K_{c}}$$

The lower the value of δ, the closer the model is to behaving like a linear model. As a rule of thumb, δ ≤ 1 indicates a good linear approximation.

***Statistical test.*** The Wilcoxon signed-rank test is a nonparametric statistical hypothesis test, which is used to test the position of a group of samples or compare the relative positions of 2 matching samples (Wilcoxon, 1945). In simple terms, it is used to evaluate the median difference between 2 paired samples. When the test data are skewed and do not meet the initial assumption of a normal distribution, the student's *t*-test does not work well (Student, 1908). In general, if the data fail to pass the Shapiro-Wilk test, a test of normality for data, the *t*-test should not be used (Shapiro and Wilk, 1965). In this case, the Wilcoxon signed-rank test would be a good alternative. In this study, we chose the Wilcoxon signed-rank test to test the significance of the difference in the RMSE_adj_ and δ values between any 2 of the 4 egg-shape equations, instead of the paired sample *t*-test, since the test data failed to pass the Shapiro-Wilk test.

### Egg-Shape Data Acquisition

**Figure 1:**
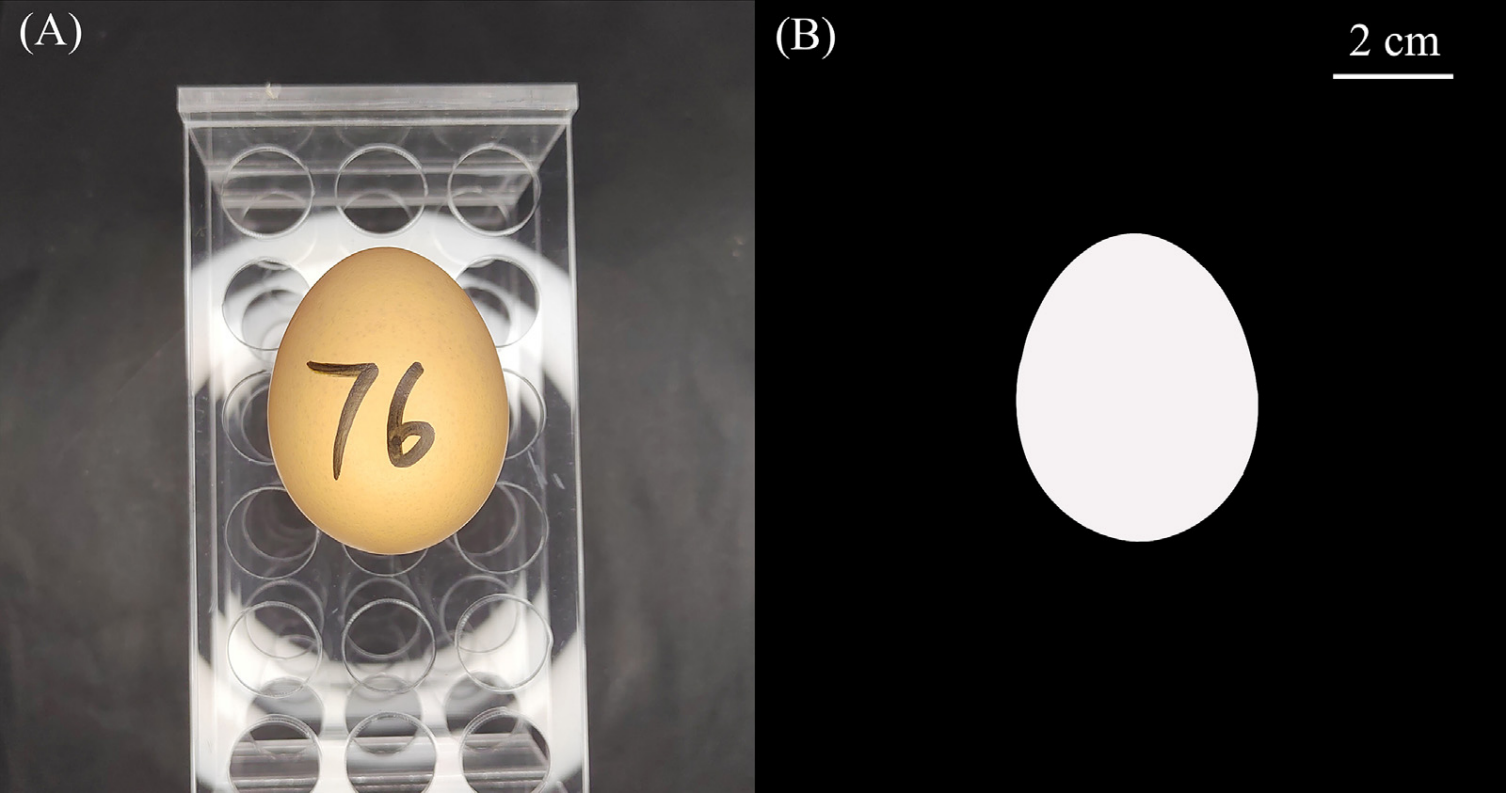
A photographed representative *G. gallus* egg (A) and its extracted 2D profile (B).

Given that *Gallus gallus domesticus* eggs are not only a commonly available foodstuff but also have a great variability in shape, 300 fresh *G. gallus* eggs were selected to supply validation data for this research. An adjustable desktop mobile phone bracket was used to fix a smart phone (Honor 60, Huawei, Dongguan, China) to capture the egg's 2D profile (see Figure 1A). Each egg was placed horizontally to make the midline of the egg parallel or approximately parallel to the desk face, with the lens of the camera facing the center of the egg. A vernier caliper was used to measure the actual length of the egg to adjust the size of the photographed egg's 2D profile to its actual size. Adobe Photoshop (version 13.0; Adobe, San Jose, CA) was used to convert the egg images into black and white bitmap files at a resolution of 600 dpi (see Figure 1B). The M-functions based on Matlab (version ≥ 2009a; MathWorks, Natick, MA) proposed by Shi et al. (2018) and Su et al*.* (2019) were used to extract egg boundary coordinate data from black and white images. Then, the “adjdata” function in the “biogeom” package (version 1.3.5; Shi et al., 2022b) based on R software (version 4.2.0; R Core Team 2022) was used to obtain 2000 approximately equidistant data points from the boundary coordinate data of each egg's 2D profile (Supplementary Table S1). In addition, photographs provided by Biggins et al. (2022) of 3 egg-shape characteristics (elongation, polar asymmetry, and pointedness) of 50 representative eggs spanning the geometries of the eggs of extant avian species were also analyzed in this study to test the generality and validity of the 4 studied egg-shape equations. The boundary coordinate data of these 50 eggs were obtained using the same protocols as described above.

### Method for Fitting the Egg-shape Equations

We digitized real eggs and obtained their 2D profiles. As mentioned above, 2000 approximately equidistant data points on each egg's 2D profile were sampled, and these data points were used to estimate the parameters of the 4 egg-shape equations using nonlinear least squares. The Nelder-Mead optimization algorithm (Nelder and Mead, 1965) was used to minimize the sum of squares of the residuals between the observed and predicted *y* values for estimating the parameters of each of the 4 equations.

## RESULTS

**Figure 2:**
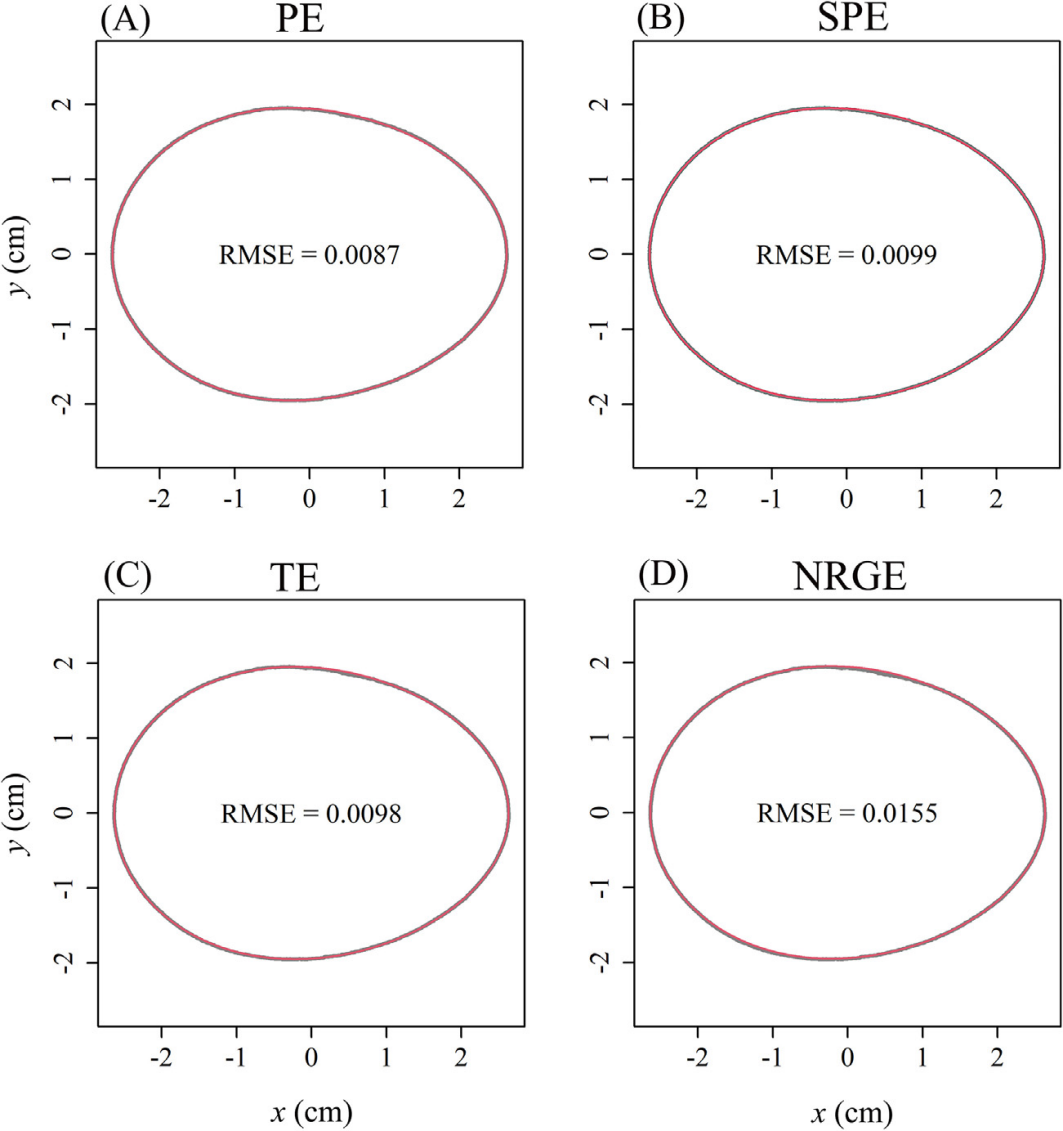
Observed (gray) and predicted (red) geometries calculated by the 4 egg-shape equations for the given egg example, i.e., the 5-parameter Preston equation (PE), the 4-parameter Preston equation (SPE), the 4-parameter Troscianko equation (TE), and the 4-parameter Narushin-Romanov-Griffin equation (NRGE). RMSE is the root-mean-square error between the observed and predicted *y*-values.

**Figure 3:**
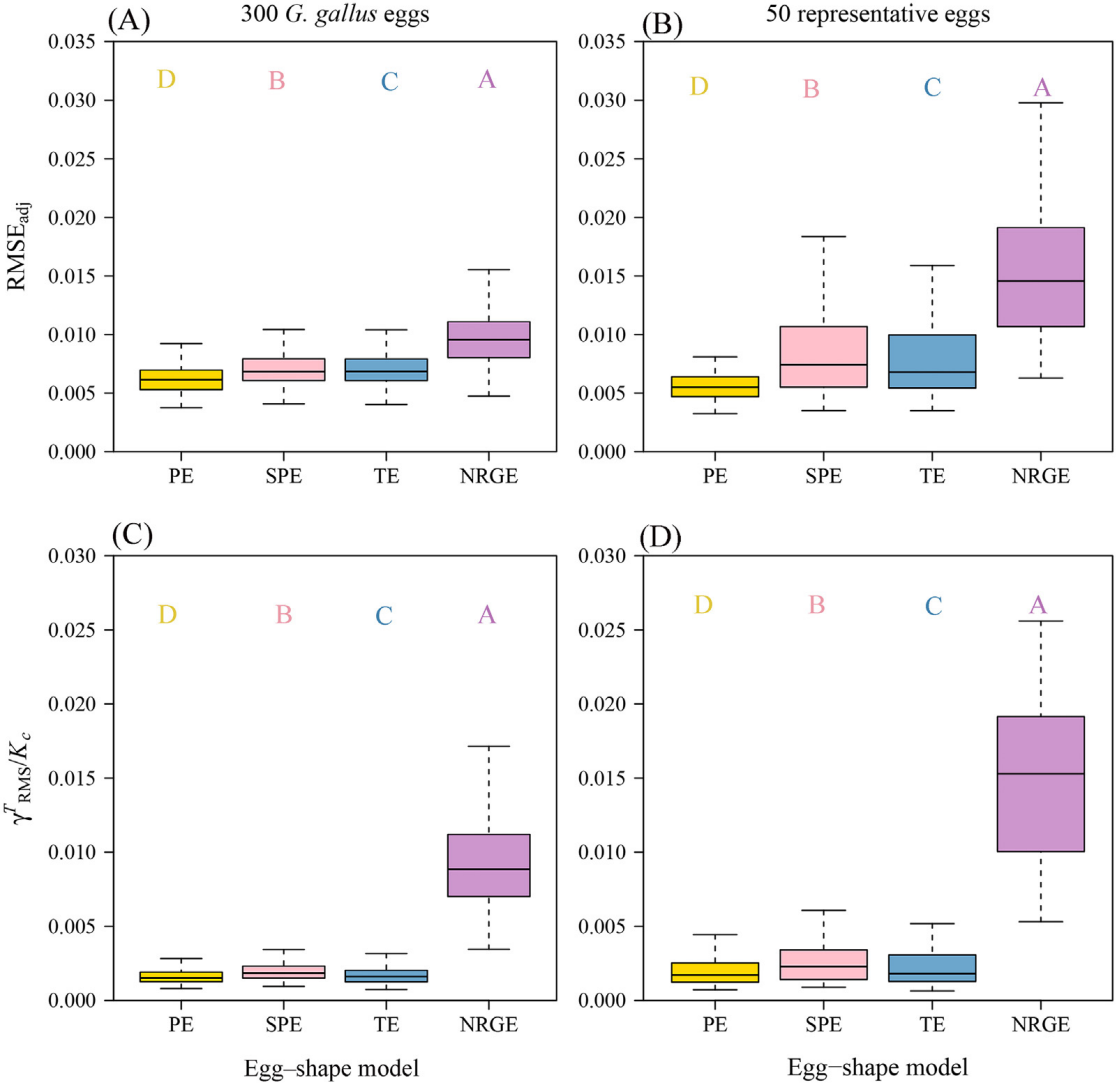
Box-and-whisker plots of the adjusted root-mean-square error (RMSE_adj_) values and the ratios (δ) of root-mean-square parameter-effects curvature to critical curvature for 300 *G. gallus* eggs (A, C), and 50 representative eggs profiles (B, D) among the 4 egg-shape equations. Uppercase letters at the top of the whiskers indicate the significance of the differences between any 2 groups of the RMSE_adj_ values or the δ values.

The RMSE_adj_ values for both the 300 *G. gallus* eggs and the 50 representative eggs fitted by the 4 egg-shape equations were all smaller than 0.035 (Supplementary Tables S2-S5 and Supplementary Tables S20-S23), which validates the 4 egg-shape equations for describing the 2D geometries of the studied eggs (Figures 2, 3A, and B). NRGE had the largest median of the RMSE_adj_ values, SPE had the second largest median, TE had the third largest median, and PE had the smallest median with all *P*-values from the Wilcoxon signed-rank test far less than 0.05 (Figures 3A, B).

The relative curvature measures (i.e., the root-mean-square intrinsic curvatures and parameter-effects curvatures) of the 4 egg-shape equations for both 300 *G. gallus* eggs and 50 representative eggs are all significantly lower than their corresponding critical curvatures (Supplementary Tables S2-S5, and Supplementary Tables S20-S23), indicating that all the 4 egg-shape equations tend to be close-to-linear (Figure 3C, and D). NRGE had the largest median of the δ values (i.e., the ratios of root-mean-square parameter-effects curvature to the critical curvature), SPE had the second largest median, TE had the third largest median, and PE had the smallest median (with all *P*-values from the Wilcoxon signed-rank test being less than 0.05).

**Figure 4:**
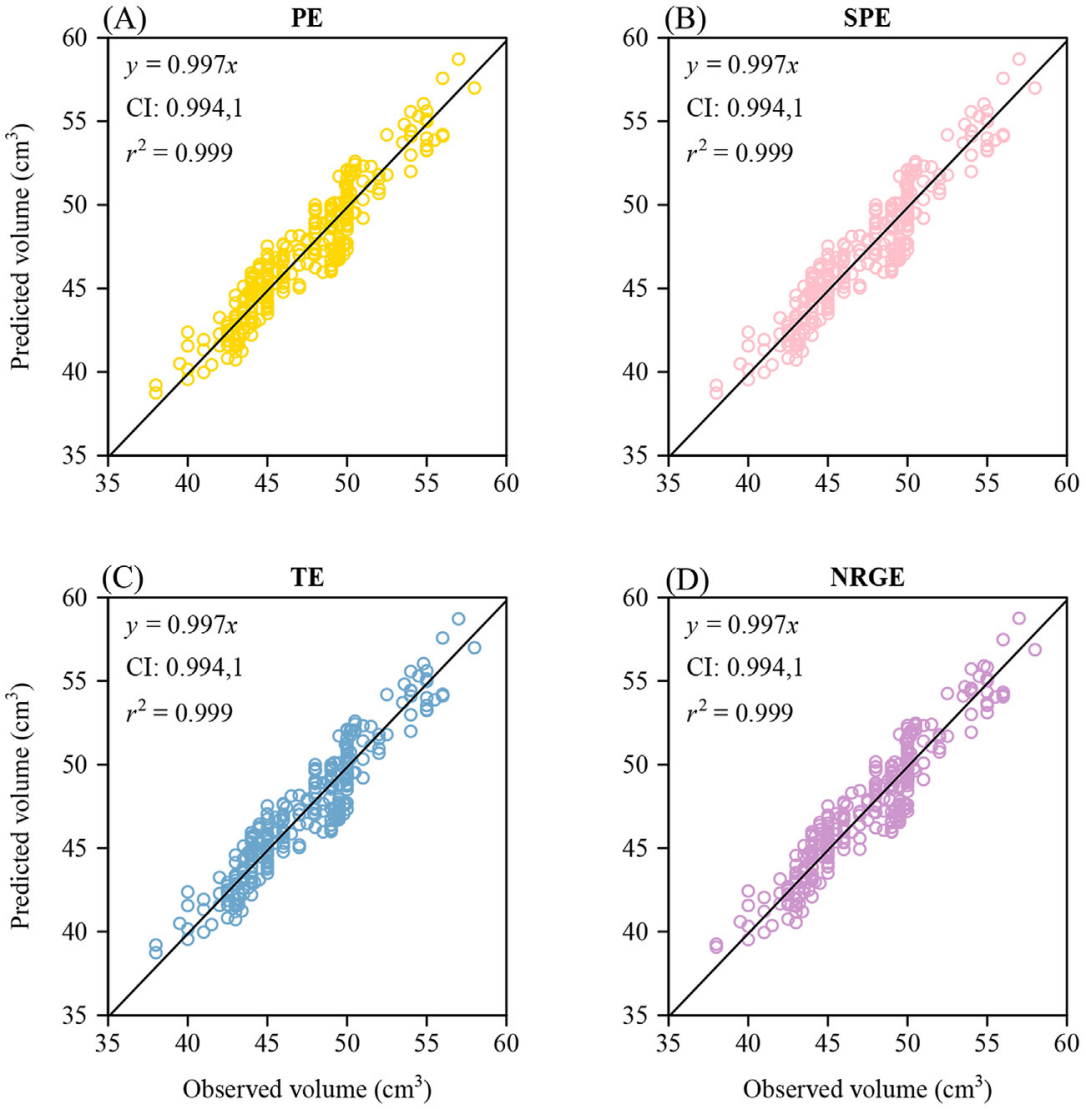
Linear fits to the predicted volume vs. the observed volume for the 4 egg-shape equations. Here, *y* denotes the volume predicted by Eq. (11) based on each of the 4 egg-shape equations, and *x* denotes the volume observed using the graduated cylinder; CI represents the 95% confidence interval of the slope; *r*^2^ represents the coefficient of determination; the straight line represents the regression line, and the open circles scattered around the regression line represent the data of the predicted volumes vs. the observed volumes of 300 *G. gallus* eggs.

Biggins et al. (2022) did not provide the empirically determined volumes (*V*) (i.e., the measured values using graduated cylinders or other approaches, e.g., using 3D laser scanners) of 50 representative eggs; therefore, we present the following results, which compare the empirically determined and predicted *V*’s based on 300 *G. gallus* eggs. The RMSE values between the predicted *V* using the formula for a solid of revolution (i.e., Eq. [11]) and the empirically determined *V* using the graduated cylinder for the 4 egg-shape equations were equal to: 1.2775 (PE), 1.2783 (SPE), 1.2781 (TE), and 1.2839 (NRGE) (Supplementary Tables S2-S5, and Supplementary Table S24). There were tiny differences in the prediction errors of *V* across the 4 equations. There was no statistically significant difference between the *V* predicted by Eq. (11) and the empirically determined *V* using the graduated cylinder, as the 95% confidence interval (**CI**) of the predicted *V* vs. the empirically determined *V* included unity (Figure 4), and the *P*-values of the paired Wilcoxon signed-rank test between the predicted *V* and the empirically determined *V* were all greater than 0.05.

We calculated the percent error (absolute value) between the *V* predicted by each of SPE, TE and NRGE and the *V* predicted by the solid of revolution formula based on PE (i.e., Eq. [11] based on Eq. [2]): the percent errors between PE and SPE ranged between 0.0000049% and 0.067% with a mean of 0.014%, the percent errors between PE and TE ranged between 0.000000448% and 0.059% with a mean of 0.0099%, and the percent errors between PE and NRGE ranged between 0.0017% and 0.85% with a mean of 0.23% (Supplementary Tables S2-S5). The results showed that the percent errors in *V* between PE and each of the remaining egg-shape equations were smaller than 1%. The percent errors in egg surface areas (*S*) calculated using Eq. (10) between PE and each of the remaining egg-shape equations were also small, with a maximum of 0.5% (Supplementary Tables S2-S5).

## DISCUSSION

### Can the Results be Extended to Other Egg Shapes?

In this study, the ability of the 4 egg-shape equations to describe the real 2D egg shapes of 300 *G. gallus* eggs was compared, with the results showing that PE had the best performance from the perspective of goodness of fit and close-to-linear behavior of nonlinearity, followed by TE, then SPE and finally NRGE. The rankings of the 4 equations were consistent for the same comparisons made using data for 50 representative eggs whose shapes span the morphological variation of extant avian eggs. The results indicate that the rankings may be general, with PE tending to perform best.

### Influence of the Sample Size on the Results of Model Comparison

The statistics we used for comparisons, such as δ (i.e., the ratio of root-mean-square parameter-effects curvature to the critical curvature) and RMSE_adj_, are affected by sample size. For example, relative curvature measures decrease with the increase in sample size (Bates and Watts, 1980; Karolczak, 1995), and there were 2,000 data points on each egg's 2D profile in the present study, which accounted for why the relative curvature measures of the egg-shape equation were so small. To clarify whether the sample size would affect the results of model comparisons, we subsequently reduced the number of data points on each egg's 2D profile from 2,000 to 1,000, 500, and 250 to examine the potential influences of these sample sizes on the model comparison. We found that the results of the RMSE_adj_-based model comparisons were affected by a small sample, as the difference between TE and SPE became statistically insignificant when the sample size was 250 (Supplementary Tables S2-S18). On the other hand, the results of the comparison of the 4 egg-shape equations based on the δ values were not affected by the change in sample size, and even when the sample size was reduced to 250, the δ values of the 4 egg-shape equations were still smaller than 0.3, although the δ values of the 4 egg-shape equations increased significantly with the reduction of the sample size (see Supplementary Figures S1 and S2, and Supplementary Tables S2-S18). This reminds us that using a sample size that is too small is likely to lead to incorrect conclusions about the validity of the equations, reflected by the RMSE_adj_ values. However, the relative curvature measures are more robust for model comparison even when the sample size is reduced to 250.

### A 5-Parameter Troscianko Equation

In this study, we found that PE exhibited the best goodness-of-fit among the 4 egg-shape equations. However, it is worth noting that PE has 5 parameters, whereas TE only has 4 parameters, and it is clear that a model with more parameters has a greater flexibility for data fitting. Thus, the additional model parameter to TE can help capture the individual-to-individual variations of avian eggs well. Since TE only has 4 parameters, we introduce an additional parameter, α_3_, to TE to render it to have the same number of parameters as PE. We denote the new model as the generalized Troscianko equation (**GTE**) .

(24) $$y_{5}=\pm a\;\text{exp}\left[\alpha_{0}+\alpha_{1}\left(\frac{x}{a}\right)+\alpha_{2}{\left(\frac{x}{a}\right)}^{2}+\alpha_{3}{\left(\frac{x}{a}\right)}^{3}\right]\sqrt{1-{\left(\frac{x}{a}\right)}^{2}}$$

We fitted the boundary coordinate data of 300 egg profiles using GTE and compared the results with those of PE. We found that the median of the RMSE_adj_ values of GTE was significantly smaller than that of PE (*P* < 0.001), and the median of the δ values of GTE was also significantly smaller than that of PE (*P* < 0.001) based on the 1-sided paired Wilcoxon signed-rank test. The data indicate that GTE works slightly better than PE for the 300 studied eggs. The results here are only applicable to the 300 *G. gallus* eggs that were studied, not to the eggs of other species. In fact, GTE did not work better than PE for the 50 eggs representing a wide variation in shape from different avian species, given that the median of the RMSE_adj_ values of GTE was significantly greater than that of PE (*P* < 0.001). However, GTE still performed better than the other 3 egg-shape equations (i.e., SPE, TE, and NRGE) for the 50 representative eggs. This means that PE is best for describing the variation in egg shape across species.

**Figure 5:**
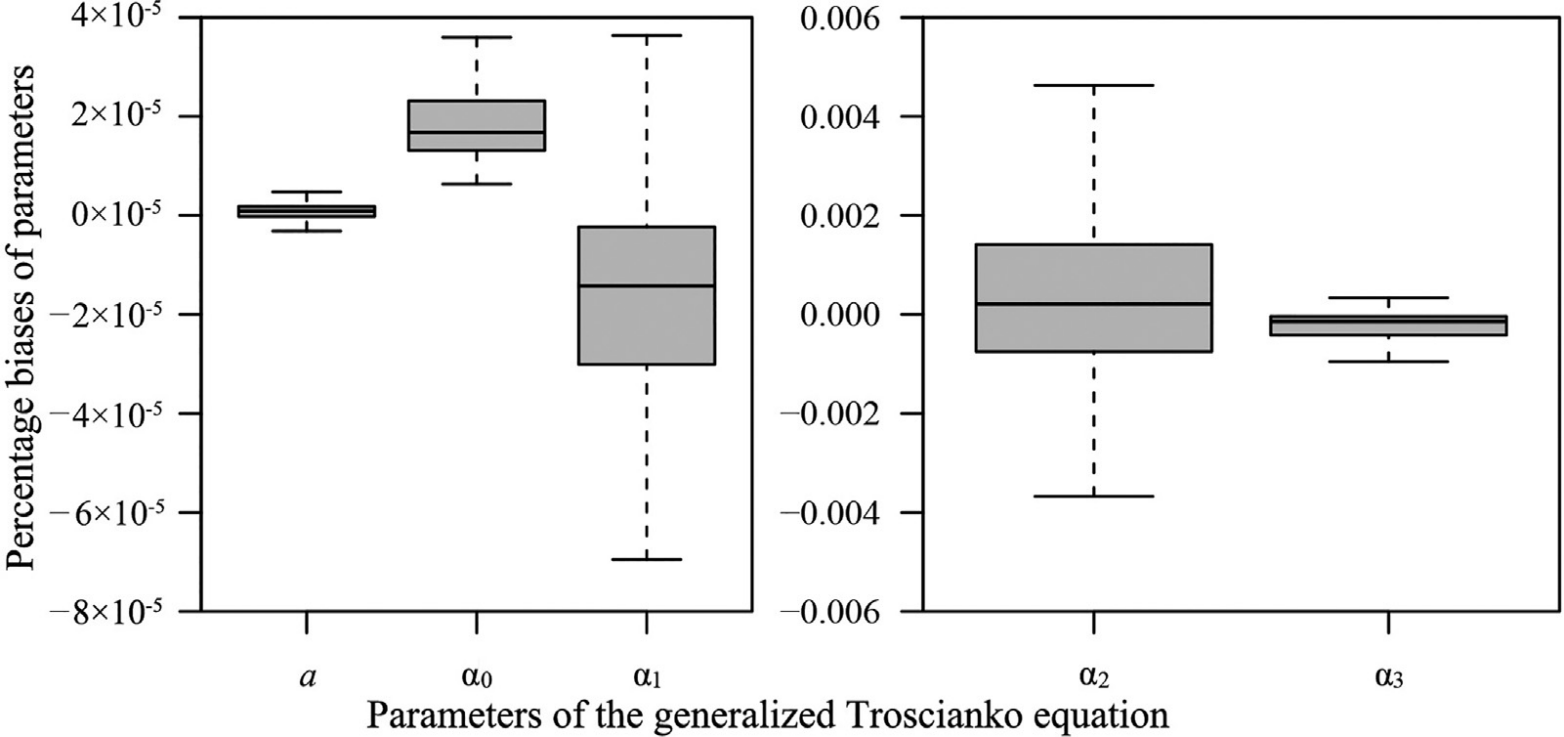
Box-and-whisker plots of the percentage bias for each parameter of the generalized Troscianko equation (GTE) based on the 300 *G. gallus* eggs.

The δ values of GTE ranged from 0.00073 to 0.0075 with a mean value of 0.0016, which showed that GTE was closer-to-linear in behavior than TE (Supplementary Table S4 vs. Supplementary Table S19). In addition, we calculated the Box's bias of each parameter of GTE (Box, 1971) using the “biasIPEC” function of the “IPEC” package (version 1.1.0), which can be used to evaluate the bias in each parameter estimate in nonlinear regression. Box's bias is used to estimate the bias in the least-squares (LS) estimators. The bias and the parameter-effects curvature are both measures of nonlinearity. However, Box's bias concerns the nonlinear behavior of each parameter of a nonlinear equation, whereas the parameter-effects curvature concerns the overall nonlinearity of the equation. When the percentage bias (= the quotient of Box's bias of a parameter and its LS estimator × 100%) is greater than a critical value (as a rule of thumb, 1%, Box [1971]), it suggests that the way in which the parameter appears in the equation should be changed to increase the nonlinear equation's approximation to that of a linear model. This change of the model form is referred to as reparameterization (Ratkowsky, 1983; Karolczak, 1995). The percentage biases of the 5 parameters of GTE for the 300 egg profile data are presented in Figure 5. Notably, the percentage biases of all 5 parameters are significantly smaller than 1%, suggesting that the least-squares estimators of these parameters have good close-to-linear properties, including the estimator of the newly added parameter.

### Calculation of the Egg Volume or Surface Area of Avian Eggs

There were negligible differences in the predicted volumes and surface areas between PE and the remaining 3 egg-shape equations, implying that any of the 4 equations can be reliably used for calculating egg volumes and surface areas. Furthermore, the volumes predicted by the 4 equations were not statistically different from the volumes determined empirically using the graduated cylinder. This observation indirectly supports the hypothesis that avian eggs can be regarded as solids of revolution (Shi et al., 2023).

## CONCLUSIONS

Based on the data of 300 *G. gallus* eggs and 50 representative avian eggs, 4 egg-shape equations, i.e., PE, SPE, TE, and NRGE, were compared. Various statistical measures, including the adjusted root-mean-square error and relative curvature measures of nonlinearity, were used to evaluate the goodness of fit and the estimation behavior of the egg-shape equations. The results indicated that PE outperformed the other equations, and that the relative curvature measures are more robust indicators for comparing the parametric equations than traditional goodness-of-fit measures. In addition, we compared the volumes predicted by the 4 egg-shape equations with the volumes empirically determined using a graduated cylinder, and the analysis revealed no significant differences between the predicted volumes of the equations. Also, the percentage errors across the 4 equations were negligible. The findings suggest that the 4 egg-shape equations that were studied are reliable and valid for calculating egg volumes. We also proposed a generalized TE with an additional parameter (denoted as GTE), and found that GTE gave a better linear approximation and better goodness of fit than the 4 studied equations for the 300 *G. gallus* eggs, but performed slightly worse than PE on 50 eggs which span a large variation in the geometries of existing avian eggs, but still outperformed the other 3 egg-shape equations. In addition, we calculated the Box's biases for the parameters of GTE, with the results demonstrating that the percentage biases of the GTE parameters were smaller than 1%, indicating close-to-linear behavior and the absence of significant bias for each parameter, including the additional parameter α_3_. The present study provides useful tools for comparing different egg-shape equations and demonstrates that PE is generally valid for fitting the 2D geometries and simulating the 3D geometries of avian eggs, and can be potentially useful in accounting for the variation of egg morphology in evolution.

## DISCLOSURES

The authors declare that they have no known competing financial interests or personal relationships that could have appeared to influence the work reported in this paper.

## Appendix Supplementary materials

Image, application 1

Image, application 2

## Footnotes

Supplementary material associated with this article can be found in the online version at doi:10.1016/j.psj.2024.104069. Supplementary Table S1. 2000 boundary coordinate data points of each of 300 *G. gallus* eggs; Supplementary Table S2. Fitted results of 300 *G. gallus* eggs' profile data using PE with a sample size of 2,000; Supplementary Table S3. Fitted results of 300 *G. gallus* eggs' profile data using SPE with a sample size of 2,000; Supplementary Table S4. Fitted results of 300 *G. gallus* eggs' profile data using TE with a sample size of 2,000; Supplementary Table S5. Fitted results of 300 *G. gallus* eggs' profile data using NRGE with a sample size of 2,000; Supplementary Table S6. Fitted results of 300 *G. gallus* eggs' profile data using GTE with a sample size of 2,000; Supplementary Table S7. Fitted results of 300 *G. gallus* eggs' profile data using PE with a sample size of 1,000; Supplementary Table S8. Fitted results of 300 *G. gallus* eggs' profile data using SPE with a sample size of 1,000; Supplementary Table S9. Fitted results of 300 *G. gallus* eggs' profile data using TE with a sample size of 1,000; Supplementary Table S10. Fitted results of 300 *G. gallus* eggs' profile data using NRGE with a sample size of 1,000; Supplementary Table S11. Fitted results of 300 *G. gallus* eggs' profile data using PE with a sample size of 500; Supplementary Table S12. Fitted results of 300 *G. gallus* eggs' profile data using SPE with a sample size of 500; Supplementary Table S13. Fitted results of 300 *G. gallus* eggs' profile data using TE with a sample size of 500; Supplementary Table S14. Fitted results of 300 *G. gallus* eggs' profile data using NRGE with a sample size of 500; Supplementary Table S15. Fitted results of 300 *G. gallus* eggs' profile data using PE with a sample size of 250; Supplementary Table S16. Fitted results of 300 *G. gallus* eggs' profile data using SPE with a sample size of 250; Supplementary Table S17. Fitted results of 300 *G. gallus* eggs' profile data using TE with a sample size of 250; Supplementary Table S18. Fitted results of 300 *G. gallus* eggs' profile data using NRGE with a sample size of 250; Supplementary Table S19. Fitted results of 50 representative eggs' profile data using GTE; Supplementary Table S20. Fitted results of 50 representative eggs' profile data using PE; Supplementary Table S21. Fitted results of 50 representative eggs' profile data using SPE; Supplementary Table S22. Fitted results of 50 representative eggs' profile data using TE; Supplementary Table S23. Fitted results of 50 representative eggs' profile data using NRGE; Supplementary Table S24. Empirically determined volume of 300 *G. gallus* eggs.

## References

[bib0001] Barta Z., Székely T. (1997). The optimal shape of avian eggs. Funct. Ecol..

[bib0002] Bates D.M., Watts D.G. (1980). Relative curvature measures of nonlinearity. J. R. Stat. Soc. B.

[bib0003] Bates D.M., Watts D.G. (1988).

[bib0004] Biggins J.D., Thompson J.E., Birkhead T.R. (2018). Accurately quantifying the shape of birds’ eggs. Ecol. Evol..

[bib0005] Biggins J.D., Montgomerie R., Thompson J.E., Birkhead T.R. (2022). Preston's universal formula for avian egg shape. Ornithology.

[bib0006] Box M.J. (1971). Bias in nonlinear estimation. J. R. Stat. Soc. B.

[bib0007] Jennrich R.I. (1969). Asymptotic properties of nonlinear least squares estimators. Ann. Math. Statist..

[bib0008] Nelder J.A., Mead R. (1965). A simplex method for function minimization. Comput. J..

[bib0009] Karolczak M. (1995). Why to calculate, when to use, and how to understand curvature measures of nonlinearity. Curr. Sep..

[bib0010] Mallock A. (1925). The shapes of birds’ eggs. Nature.

[bib0011] Narushin V.G., Romanov M.N., Griffin D.K. (2021). Egg and math: introducing a universal formula for egg shape. Ann. N. Y. Acad. Sci..

[bib0012] Nishiyama Y. (2012). The mathematics of egg shape. Int. J. Pure Appl. Math..

[bib0013] Paganelli C.V., Olszowka A., Ar A. (1974). The avian egg: surface area, volume, and density. Condor.

[bib0014] Petrović M., Obradović M., Mijailović R. (2011). Proc. 4th Int. Conf. Eng. Graphics Design.

[bib0015] Preston F.W. (1953). The shapes of birds’ eggs. Auk.

[bib0016] R Core Team. 2022. R: A language and environment for statistical computing. R Foundation for statistical computing, Vienna, Austria. Website: https://www.r-project.org/ [Accessed June 1, 2022].

[bib0017] Ratkowsky D.A. (1983).

[bib0018] Ratkowsky D.A., Reddy G.V.P. (2017). Empirical model with excellent statistical properties for describing temperature-dependent developmental rates of insects and mites. Ann. Entomol. Soc. Am..

[bib0019] Regueiro-Picallo M., Naves J., Anta J., Puertas J., Suárez J. (2016). Experimental and numerical analysis of egg-shape sewer pipes flow performance. Water.

[bib0020] Shapiro S.S., Wilk M.B. (1965). An analysis of variance test for normality (complete samples). Biometrika.

[bib0021] Shi P., Chen L., Quinn B.K., Yu K., Miao Q., Guo X., Lian M., Gielis J., Niklas K.J. (2023). A simple way to calculate the volume and surface area of avian eggs. Ann. N. Y. Acad. Sci..

[bib0022] Shi P., Gielis J., Niklas K.J. (2022). Comparison of a universal (but complex) model for avian egg shape with a simpler model. Ann. N. Y. Acad. Sci..

[bib0023] Shi P., Gielis J., Quinn B.K., Niklas K.J., Ratkowsky D.A., Schrader J., Ruan H., Wang L., Niinemets Ü. (2022). ‘biogeom’: An R package for simulating and fitting natural shapes. Ann. N. Y. Acad. Sci..

[bib0024] Shi P., Ratkowsky D.A., Li Y., Zhang L., Lin S., Gielis J. (2018). General leaf-area geometric equation exists for plants—evidence from the simplified Gielis equation. Forests.

[bib0025] Stoddard M.C., Sheard C., Akkaynak D., Yong E.H., Mahadevan L., Tobias J.A. (2019). Evolution of avian egg shape: underlying mechanisms and the importance of taxonomic scale. Ibis.

[bib0026] Stoddard M.C., Yong E.H., Akkaynak D., Sheard C., Tobias J.A., Mahadevan L. (2017). Avian egg shape: Form, function, and evolution. Science.

[bib0027] Student (1908). The probable error of a mean. Biometrika.

[bib0028] Su J., Niklas K.J., Huang W., Yu X., Yang Y., Shi P. (2019). Lamina shape does not correlate with lamina surface area: An analysis based on the simplified Gielis equation. Glob. Ecol. Conserv..

[bib0029] Thompson D.W. (1917).

[bib0030] Todd P.H., Smart I.H.M. (1984). The shape of birds’ eggs. J. Theor. Biol..

[bib0031] Troscianko J. (2014). A simple tool for calculating egg shape, volume and surface area from digital images. Ibis.

[bib0032] Wilcoxon F. (1945). Individual comparisons by ranking methods. Biom. Bull..

[bib0033] Zlatev Z., Georgieva-Nikolova M., Lukanov H. (2022). An algorithm for obtaining 3D egg models from visual images. Appl. Sci..

