# Supplementary material for: Comparison of egg-shape equations using relative curvature measures of nonlinearity
Source: Poult Sci. 2024 Jul 10;103(10):104069. doi: 10.1016/j.psj.2024.104069 (PMC11338082; doi:10.1016/j.psj.2024.104069)
Supplement: Supplementary file 1 [file mmc1.docx]

Supplementary figure legends

FIGURE S1. Box-and-whisker plots of the adjusted root mean square error (RMSE_adj_) values for 300 *G. gallus* egg profiles with four sample sizes for the four egg-shape equations. N represents the sample size (i.e., the number of boundary coordinate data points extracted from the egg’s 2D profile). Different uppercase letters at the top of the whiskers indicate a significant difference (*p* < 0.05) between any pair of RMSE_adj_ values.

FIGURE S2. Box-and-whisker plots of the ratios (δ’s) of the parameter-effects curvature to the critical curvature for 300 *G. gallus* egg profiles with four sample sizes for the four egg-shape equations. N represents the sample size (i.e., the number of boundary coordinate data points extracted from the egg’s 2D profile). Different uppercase letters at the top of the whiskers indicate a significant difference (*p* < 0.05) between any pair of δ values.


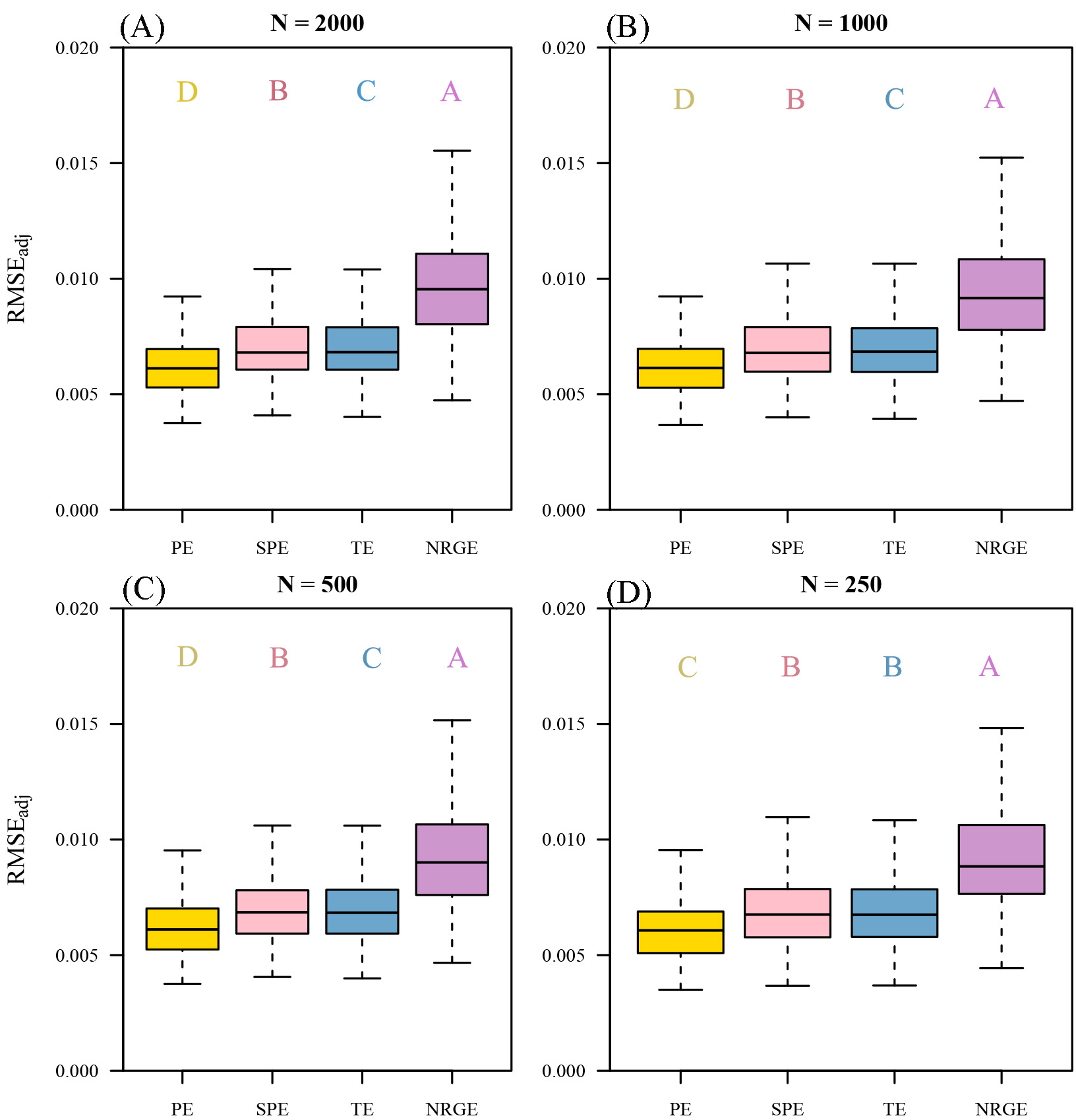


Figure S1


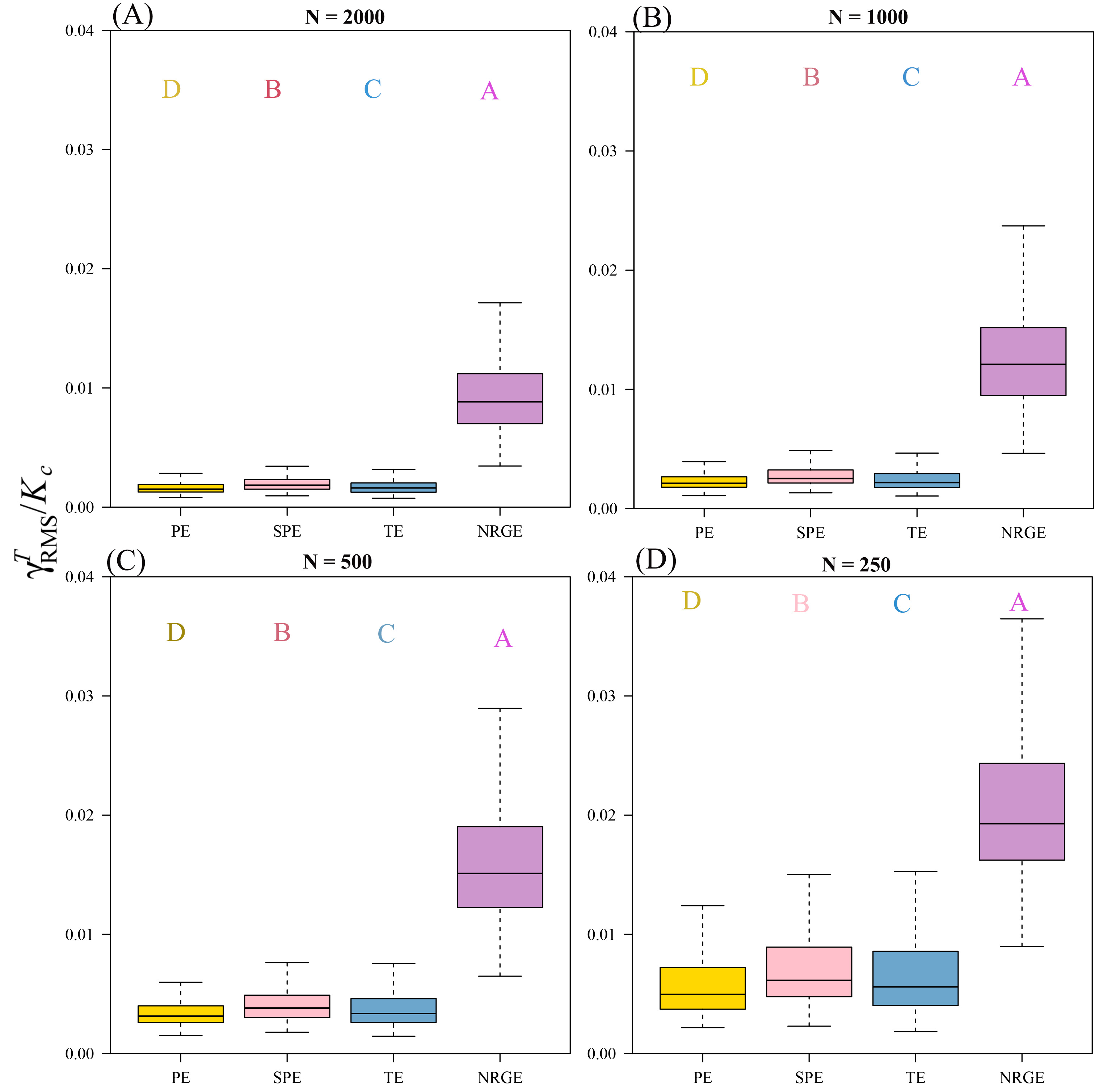


Figure S2
